# Performance of acute respiratory distress syndrome definitions in a high acuity paediatric intensive care unit

**DOI:** 10.1186/s12931-021-01848-z

**Published:** 2021-09-29

**Authors:** Michelle Rudolph, Jefta van Dijk, Pauline de Jager, Sandra K. Dijkstra, Johannes G. M. Burgerhof, Robert G. T. Blokpoel, Martin C. J. Kneyber

**Affiliations:** 1 Division of Paediatric Critical Care Medicine, Department of Paediatrics, Beatrix Children’s Hospital, University Medical Center Groningen, University of Groningen, Huispost CA62, P.O. 30.001, 9700 RB Groningen, The Netherlands; 2 Department of Epidemiology, University Medical Center Groningen, University of Groningen, Groningen, The Netherlands; 3 Critical Care, Anaesthesiology, Peri-Operative & Emergency Medicine (CAPE), University Medical Center Groningen, University of Groningen, Groningen, The Netherlands

**Keywords:** Acute respiratory distress syndrome, Paediatric acute lung injury consensus conference, American–European consensus conference, Berlin definition, PICU, Mortality, Predictive value, Mechanical ventilation, Paediatric

## Abstract

**Background:**

For years, paediatric critical care practitioners used the adult American European Consensus Conference (AECC) and revised Berlin Definition (BD) for acute respiratory distress syndrome (ARDS) to study the epidemiology of paediatric ARDS (PARDS). In 2015, the paediatric specific definition, Paediatric Acute Lung Injury Consensus Conference (PALICC) was developed. The use of non-invasive metrics of oxygenation to stratify disease severity were introduced in this definition, although this potentially may lead to a confounding effect of disease severity since it is more common to place indwelling arterial lines in sicker patients. We tested the hypothesis that PALICC outperforms AECC/BD in our high acuity PICU, which employs a liberal use of indwelling arterial lines and high-frequency oscillatory ventilation (HFOV).

**Methods:**

We retrospectively collected data from children < 18 years mechanically ventilated for at least 24 h in our tertiary care, university-affiliated paediatric intensive care unit. The primary endpoint was the difference in the number of PARDS cases between AECC/BD and PALICC. Secondary endpoints included mortality and ventilator free days. Performance was assessed by the area under the receiver operating characteristics curve (AUC-ROC).

**Results:**

Data from 909 out of 2433 patients was eligible for analysis. AECC/BD identified 35 (1.4%) patients (mortality 25.7%), whereas PALICC identified 135 (5.5%) patients (mortality 14.1%). All but two patients meeting AECC/Berlin criteria were also identified by PALICC. Almost half of the cohort (45.2%) had mild, 33.3% moderate and 21.5% severe PALICC PARDS at onset. Highest mortality rates were seen in patients with AECC acute lung injury (ALI)/mild Berlin and severe PALICC PARDS. The AUC-ROC for Berlin was the highest 24 h (0.392 [0.124–0.659]) after onset. PALICC showed the highest AUC-ROC at the same moment however higher than Berlin (0.531 [0.345–0.716]). Mortality rates were significantly increased in patients with bilateral consolidations (9.3% unilateral vs 26.3% bilateral, *p* = 0.025).

**Conclusions:**

PALICC identified more new cases PARDS than the AECC/Berlin definition. However, both PALICC and Berlin performed poorly in terms of mortality risk stratification. The presence of bilateral consolidations was associated with a higher mortality rate. Our findings may be considered in future modifications of the PALICC criteria.

**Supplementary Information:**

The online version contains supplementary material available at 10.1186/s12931-021-01848-z.

## Background

For long, paediatric critical care practitioners have adopted the American European Consensus Conference (AECC) definition of acute respiratory distress syndrome (ARDS) established in 1994 and its 2012 revised definition, the Berlin definition, to describe and study the epidemiology of ARDS in children [1–3]. Using this adult-based definition, the incidence of paediatric ARDS (PARDS) varies between 2 and 10 cases per 100,000 per year [4–11]. Despite this apparent low incidence, PARDS is associated with significant but at the same time variable mortality rates. Different observational and epidemiological studies of children with PARDS indicate mortality rates of 15–75% [12]. Although PARDS may differ from ARDS in adults in terms of epidemiology and triggering factors, the major limitation of both the AECC and Berlin definition is the use of invasive metrics of oxygenation (i.e., the PaO_2_/FiO_2_ ratio). Use of indwelling arterial lines is not common in children, especially in less severe disease, increasing the likelihood of underestimating the true prevalence of PARDS [5, 13].

In 2015, the Paediatric Acute Lung Injury Consensus Conference (PALICC) definition of PARDS was published, addressing the issues related to the differences between children and adults [14]. PALICC definition mirrors the Berlin definition except for two criteria. First, instead of the PaO_2_/FiO_2_ (PF) ratio, PALICC makes use of the oxygenation index (OI) or the oxygenation saturation index (OSI) when indwelling arterial lines are absent as oxygenation metrics for severity stratification in intubated patients. Second, PALICC does not require the presence of bilateral consolidations on chest radiograph. The AECC definition differs also in severity stratification and only distinguishes two severity levels, where the Berlin definition and PALICC distinguish three severities.

Better performance of PALICC over the adult-based definitions was demonstrated in a prospective, observational cross-sectional study in 145 international paediatric intensive care units (PICU) [15]. In this study, applying the PALICC definition identified more patients with high mortality risk than the Berlin definition and appeared to adequately stratify mortality risk when applied 6 h after PARDS diagnosis. However, less than half of the study population had invasive metrics of oxygenation upon PARDS diagnosis making it difficult to understand the confounding effect of disease severity as it is probably more common to put indwelling arterial lines in sicker patients. Therefore, we sought to explore the performance of PALICC compared with the Berlin definition in our high acuity PICU with a liberal use of indwelling arterial lines in invasively ventilated patients and use of high-frequency oscillatory ventilation (HFOV) by comparing the number of PARDS cases identified by both definitions. Secondary objectives included the difference between PALICC, Berlin and AECC definition in terms of mortality discrimination and PARDS severity trajectory during the first 48 h.

## Methods

We retrospectively collected data from all children > 7 days (for cardiac patients, they can be admitted from birth) to < 18 years admitted to the PICU of the Beatrix Children’s Hospital between January 2014 and December 2016 who required invasive mechanical ventilation (MV) > 24 h. Patients with chronic lung disease were included if they met standard diagnostic criteria and had an acute deterioration in oxygenation from their baseline. Patients with uncorrected cyanotic heart disease, home ventilation and those on extra-corporeal life support (ECLS) were not studied. The Institutional Review Board waived the need for informed consent.

### Screening of patients

Data was extracted from the patient’s paper and electronic health records. First, two reviewers (MR and JvD) evaluated chest radiographs for the presence of unilateral or bilateral consolidations in the period 24 h before admission to the PICU and the first 8 days of admission (see Additional file 1). If deemed present, an independent senior paediatric critical care physician (RB or MK) confirmed or refuted the presence of consolidations. When patients met the radiographic criteria for ARDS, we screened for hypoxaemia (i.e. PF < 300 or OI ≥ 4 (or OSI > 5 when OI was unavailable)) within 24 h of the chest radiograph [5]. Echocardiograms were not routinely performed in our unit, hence we assumed that left ventricular (LV) dysfunction was not present unless clinically documented and confirmed by echocardiography.

### Definitions for PARDS

We applied published AECC and Berlin definitions [2, 3]. Briefly, criteria for meeting the AECC definition included (a) identification of known trigger, (b) bilateral consolidations on the chest radiograph, (c) absence of LV dysfunction and (d) PF < 300. For the Berlin definition, patients had to meet the same criteria at a minimal PEEP of 5 cm H_2_O and mild LV dysfunction were included. For both definitions, initial PARDS severity was classified by the PF ratio closest to the confirmatory chest radiograph. The closest PF ratio 24 and 48 h ± 6 h after the initial ratio was used to classify the 24 and 48 h PARDS severity. Using the Berlin definition, PARDS severity was defined as mild (200 < PF ≤ 300 mmHg), moderate (100 < PF ≤ 200 mmHg) and severe (PF ≤ 100 mmHg). The AECC definition discriminated acute lung injury (ALI) (200 < PF ≤ 300 mmHg) and ARDS (PF ≤ 200 mmHg).

Patients met PALICC criteria when (a) a trigger was identified, (b) there were ≥ 1 consolidations on chest radiograph, (c) LV dysfunction was ruled out and (d) OI ≥ 4 or OSI ≥ 5 when no PaO_2_ was available. Initial PARDS severity was classified by OI ≥ 4 or OSI ≥ 5 closest to the confirmatory chest radiograph. The closest OI or OSI 24 and 48 h ± 6 h after the initial ratio was used to classify the 24 and 48-h PARDS severity. PARDS severity was stratified as mild (4 ≤ OI < 8 or 5 ≤ OSI < 7.5), moderate (8 ≤ OI < 16 or 7.5 ≤ OSI < 12.3) and severe (OI ≥ 16 or OSI ≥ 12.3).

### Additional data collection

For each patient, we collected demographical and clinical data including age, gender, admission diagnosis, duration of MV, MV mode, length of PICU stay and survival status. Ventilator free days (VFD) was calculated by subtracting MV duration of 28 and deceased patients were assigned with 0 VFD [16]. MV mode was stratified in three categories; HFOV for 48 h, combination of HFOV and conventional mechanical ventilation (CMV) during the first 48 h, CMV for 48 h. Disease severity was assessed using the 24-h Paediatric Risk of Mortality (PRISM) III score as proxy [17]. Although there was no clinical algorithm dictating its use, indwelling catheters are routinely inserted and arterial blood gasses (ABGs) are done at least 4 times a day early in the disease trajectory. The continuous distending pressure (CDP) was used instead of the mean airway pressure (mPaw) when patients were on the oscillator.

### Study endpoints

The primary endpoint was the difference in the number of PARDS cases between AECC, Berlin and PALICC. Secondary endpoints were the difference in mortality rates, MV duration, PICU length of stay and ventilator free days (VFD).

### Statistical analysis

For continuous patient characteristics and outcome data, descriptive statistics were used where mean (± SD) was used for normally distributed data, which was assessed by the Kolmogorov–Smirnov test, and median (IQR) were used for non-normal distributed data. Mortality rates as a function of PARDS severity, MV mode and chest consolidations were tested using the χ^2^ or Fisher’s Exact test. Receiver operating characteristics curves (ROC) were constructed to evaluate the mortality discrimination by calculating the area under the curve (AUC) with 95% confidence intervals (CI). Kruskal Wallis tests were used to test differences in length of MV, VFD and PICU stay between PARDS severity strata and MV mode. Mann–Whitney U tests were used to test the differences in length of MV, VFD and PICU stay between unilateral and bilateral consolidations. *p* values < 0.05 were accepted as statistically significant. All statistical analyses were performed with SPSS 24 (IBM, Chicago, Ill, USA).

## Results

**Fig. 1:**
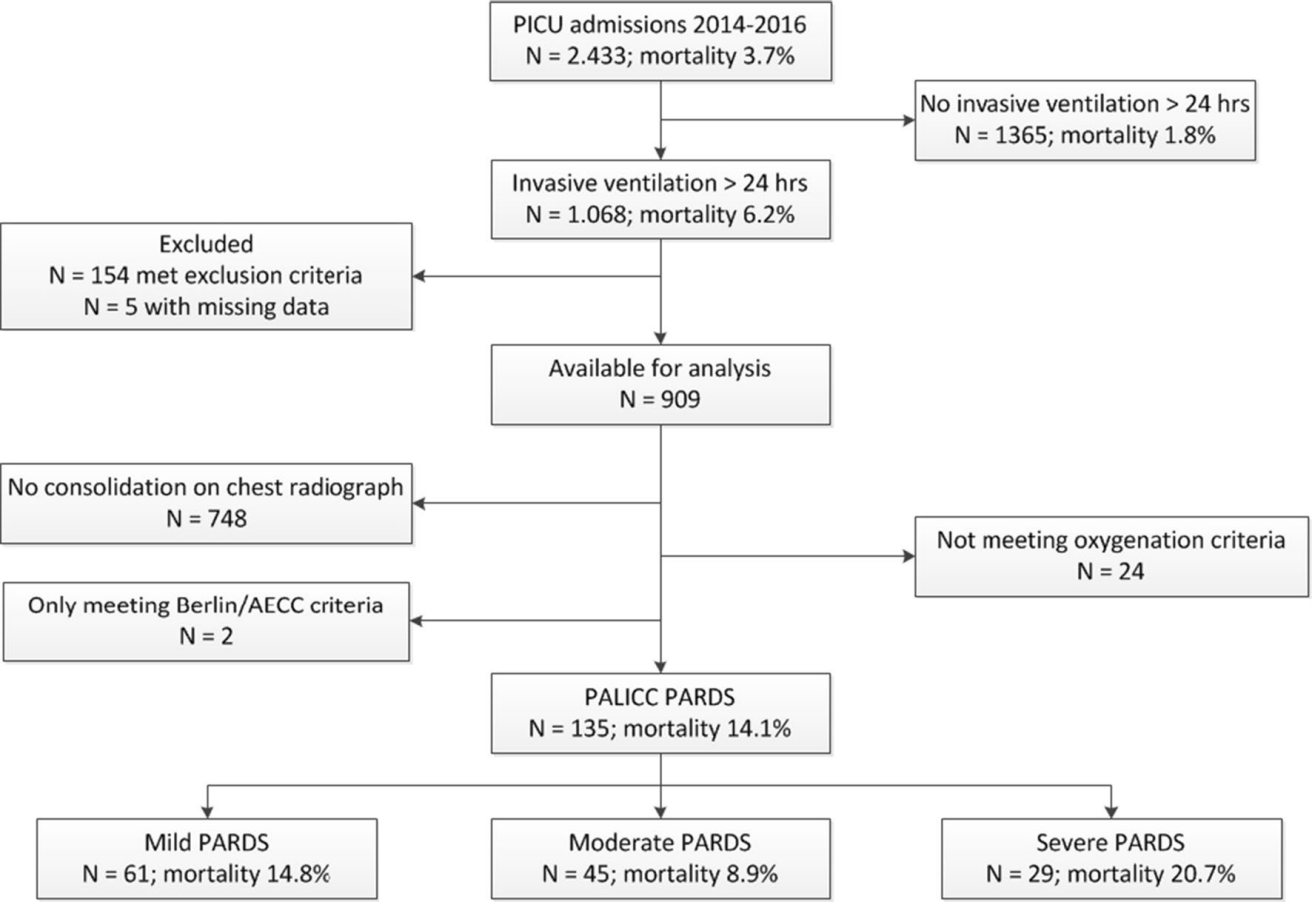
Flow diagram of the study population

**Fig. 2:**
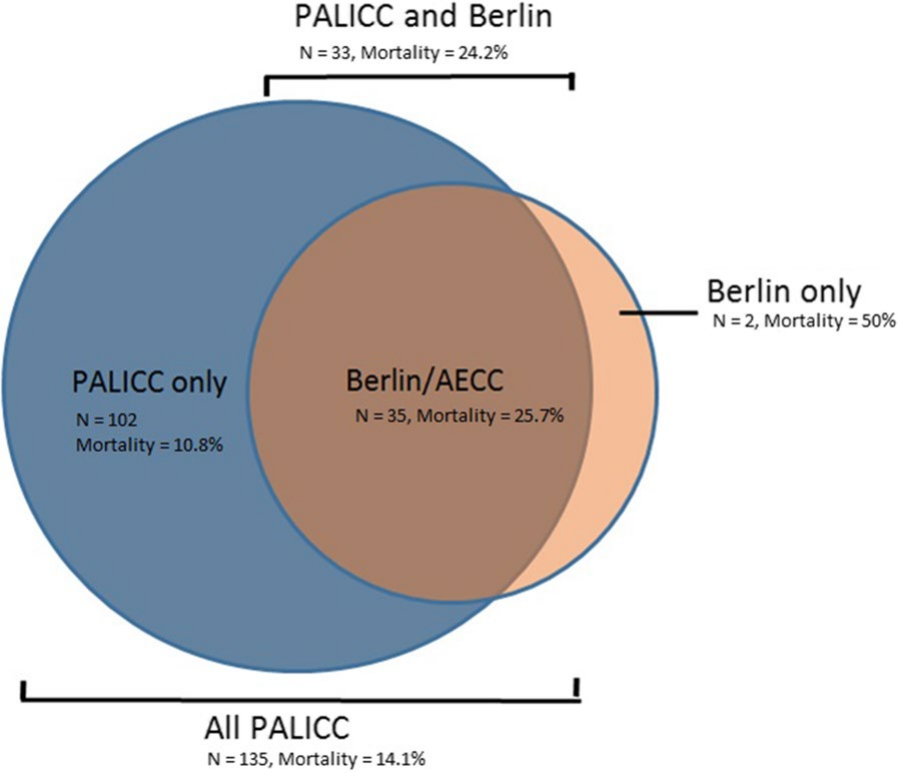
Distribution of subjects meeting ARDS criteria stratified by ARDS definition. The blue area represents 102 subjects only meeting PALICC criteria, the dark orange area represents 33 subjects meeting PALICC and Berlin/AECC criteria. The orange area represents the two subjects only meeting Berlin/AECC criteria

A total of 2.433 patients (mortality 3.7%) were admitted to the PICU during the study period, of whom N = 1.068 (43.9%) were ventilated > 24 h (Fig. 1). Of those, N = 154 patients met the exclusion criteria and five had missing data, yielding data from N = 909 patients available for analysis. There were no cases with mild LV dysfunction which made the identification by the Berlin definition equal to the AECC definition which identified N = 35 patients (1.4% of all PICU admissions and 3.3% of those ventilated > 24 h) with a mortality of 25.7% (Fig. 2). All but two patients meeting AECC/Berlin criteria were also identified by PALICC. One-hundred-and-thirty-five patients (5.5% of all PICU admissions and 12.6% of those ventilated > 24 h) met PALICC criteria, of whom N = 102 were not identified by AECC/Berlin. Ninety-five percent of those had invasive metrics for oxygenation available. The mortality rate was 14.1%. Patient characteristics, admission diagnosis and outcomes were well-balanced between the AECC/Berlin and PALICC PARDS definition (Table 1).

**Table 1:** Patient characteristics of cohorts meeting ARDS criteria

	PALICC	Berlin/AECC	*p value*
N	135	35	
Male gender, *%*	59.3	60	*0.937*
Age, *median in days (IQR)*	380 (61;1917)	770 (137;2310)	*0.179*
PRISM-II, *median (IQR)*	14 (10;19)	14 (12;23)	*0.378*
PRISM-III, *median (IQR)*	4 (1;8)	5 (1;8)	*0.811*
PIM, *median (IQR)*	− 2.505 (− 3.249;− 1.940)	− 2.380 (− 2.660;− 1.530)	*0.064*
PIM-II, *median (IQR)*	− 3.537 (− 4.382;− 2.487)	− 2.879 (− 4.210;− 1.587)	*0.052*
Mortality, *%*	14.1	25.7	*0.098*
Receiving HFOV at onset, *n (%)*	18 (13.3)	4 (11.4)	*0.765*
Timing			
Between chest radiographs and meeting criteria, *median in minutes (IQR)*	47 (28.5;143)	39.5 (20.5;111.75)	*0.366*
Intubation cause			
Pneumonia, *n (%)*	78 (58)	18 (51)	*0.409*
Sepsis, *n (%)*	14 (10)	6 (17)	
Post resuscitation, *n (%)*	11 (8)	5 (14)	
Post cardiac surgery, *n (%)*	9 (7)	0 (0)	
High energetic trauma, *n (%)*	4 (3)	0 (0)	
Status epilepticus, *n (%)*	2 (1)	1 (3)	
Liver failure/transplantation, *n (%)*	1 (< 1)	1 (3)	
Others, *n (%)*	16 (12)	4 (11)	

No significant differences were found between the cohort meeting PALICC criteria and the cohort meeting Berlin/AECC criteria

**Fig. 3:**
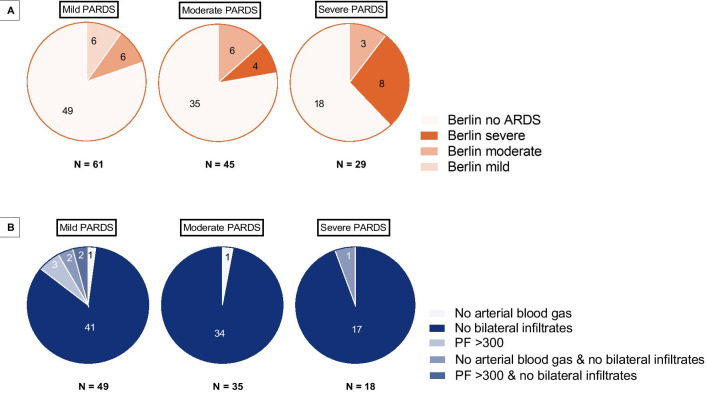
**A** Differences in severity stratification between PALICC and Berlin definition. Most of the 61 subjects meeting PALICC criteria for mild PARDS did not meet Berlin/AECC criteria, 6 also met criteria of mild ARDS by Berlin and ALI byAECC and 6 met criteria of moderate ARDS by Berlin and ARDS by AECC. Of the 45 subjects meeting PALICC criteria for moderate PARDS by PALICC only 6 subject met criteria for moderate ARDS by Berlin and 4 subjects met criteria for severe ARDS by the Berlin definition. Of the 29 subjects meeting PALICC criteria for severe PARDS 8 also met criteria for severe ARDS by the Berlin definition and 3 met Berlin criteria for moderate ARDS. **B** Reasons PALICC PARDS subjects are not identified as ARDS by Berlin/AECC. 49 subjects with mild PARDS by PALICC were not identified by Berlin/AECC, mainly due to lack of bilateral consolidations (dark blue). For the 35 subjects that were not identified by Berlin/AECC with moderate PARDS and the 18 subjects with severe PARDS a same pattern was seen

Figure 3A graphically summarizes PARDS severity at onset. Almost half of the cohort (45.2%) had mild, 33.3% moderate and 21.5% severe PALICC PARDS at onset. AECC/Berlin identified 19.7% of patients with PALICC mild PARDS (Fig. 3A). For patients with moderate PALICC PARDS this was 22.2% and for severe PALICC PARDS 37.9%. Reasons for not identifying patients with PARDS included absence of bilateral consolidations (83.6% mild, 97.1% moderate, 94.4% severe) or invasive metrics of oxygenation (2% mild, 2.9% moderate, 0% severe) (Fig. 3B).

Table 2 summarizes Berlin and PALICC PARDS severity strata assessed at onset, 24 and 48 h later. There was no direct relationship with mortality for both definitions. Highest mortality rates were seen in patients with mild AECC/Berlin and severe PALICC PARDS. The AUC-ROC for AECC/Berlin was the highest at onset (0.391 [0.176–0.606] and 24 h (0.392 [0.124–0.659]). PALICC showed a higher AUC-ROC and was also the highest 24 h after onset (0.531(0.345–0.716)). There was no difference in duration of MV or length of PICU stay among patients meeting only Berlin criteria (Table 3). Similar observations were made for PALICC PARDS at onset. However, 24 h after onset MV duration, PICU length of stay and VFD differed significantly and 48 h after onset a similar difference was seen for the MV duration and VFD.

**Table 2:** Performance of the Berlin and PALICC definition on predicting mortality

	Berlin			PALICC		
	Mild	Moderate	Severe	Mild	Moderate	Severe
Onset						
Number of patients	8	15	12	61	45	29
PICU mortality	37.5	26.7	16.7	14.8	8.9	20.7
AUC-ROC	0.391 (0.176–0.606)			0.522 (0.371–0.673)		
* p-value*	*0.336*			*0.757*		
At 24 h						
Number of patients	5	20	1	45	34	26
PICU mortality	40.0	20.0	0	13.3	5.9	19.2
AUC-ROC	0.392 (0.124–0.659)			0.531 (0.345–0.716)		
* p-value*	*0.429*			*0.723*		
At 48 h						
Number of patients	13	9	4	36	34	20
PICU mortality	38.5	11.1	0	8.3	11.8	10.0
AUC-ROC	0.267 (0.060–0.473)			0.529 (0.336–0.722)		
* p-value*	*0.088*			*0.778*		

At onset the Berlin stratification had an AUC-ROC of 0.391 (0.176–0.606), 24 h after onset it was 0.392 (0.124–0.659) and 48 h after onset it was 0.267 (0.060–0.473). On the right the PALICC definition is represented and shows an AUC-ROC of 0.522 (0.371–0.673) for mortality at onset, 0.531 (0.345–0.716) 24 h after onset and 0.529 (0.336–0.722) 48 h after onset

**Table 3:** Performance of the Berlin and PALICC definition on predicting MV duration, PICU length of stay and VFD

	Berlin				PALICC			
	Mild	Moderate	Severe	*p value*	Mild	Moderate	Severe	*p value*
Onset								
Number of patients	8	15	12		61	45	29	
Duration of MV	7.5 (3.75;12)	9 (5;13)	10.5 (7.25;14.75)	*0.454*	8 (4.5;13)	8 (5;12)	10 (5.5;13)	*0.663*
PICU length of stay	8.5 (3;16.25)	10 (5;13)	11.5 (8.25;19.75)	*0.289*	8 (4;17.5)	8 (7;14)	10 (5.5;14)	*0.696*
VFD	17 (0;21.25)	19 (0;21)	15.5 (2.5;20.75)	*0.906*	19 (7;23)	20 (15.5;22.5)	17 (9;20)	*0.102*
At 24 h								
Number of patients	5	20	1		45	34	26	
Duration of MV	6 (3.5;10)	10 (8;17.25)	14	*0.060*	7 (5;10)	9.5 (6.75;15)	11 (8;14.25)	0.000*
PICU length of stay	10 (3.5;11.5)	10.5 (9;21.5)	14	*0.320*	7 (5;11)	10.5 (7.75;19.25)	11.5 (8.75;16.5)	0.002*
VFD	21 (0;24.5)	17.5 (0;20)	14	*0.650*	20 (16;23)	18.5 (12.75;21.25)	16 (3;19.25)	0.006*
At 48 h								
Number of patients	13	9	4		36	34	20	
Duration of MV	9 (6;20)	12 (7.5;16.5)	9.5 (9;10.75)	*0.751*	8 (5.25;10.75)	10.5 (7;15)	11 (8.25;15.75)	0.008*
PICU length of stay	10 (8;22)	15 (9;21)	10 (9.25;10.75)	*0.492*	8.5 (7;14)	10.5 (7.75;19.25)	11.5 (9.25;20.25)	*0.118*
VFD	15 (0;21.5)	14 (5;20.5)	18.5 (17.25;19)	*0.897*	20 (16.25;22.75)	8 (17;21)	17 (12.25;19.75)	0.027*

On the left the Berlin/AECC definition is presented, on the right the PALICC criteria. Significant differences were found in MV duration and VFD when stratified by PALICC 24 h and 48 h after onset and in admission duration when stratified by PALICC 24 h after onset. Kruskal–Wallis test was used, α = 0.05

*Indicates significant differences

Ninety-seven patients (71.9%) with PALICC PARDS had unilateral chest radiograph consolidations. Unilateral consolidations was associated with a lower mortality compared to bilateral consolidations (9.3% vs. 26.3%. *p* = *0.025*) (Table 4). A similar difference was found for the VFD (20 [IQR 14.5–23] vs 16.5 [IQR 0–21], *p* = *0.019*) and PICU length of stay stay (8 days ([QR 5–13] vs 10 days [IQR: 7.75–19.25], *p* = *0.03*). Duration of MV (8 days [IQR 5–11.5] vs 9 days [IQR: 6.75–14.25]) did not differ significantly (*p* = *0.054*).

**Table 4:** Differences in outcome measures for unilateral vs bilateral consolidations in PALICC population

	Unilateral	Bilateral	*p-value*
PICU mortality, *in %*	9.3%	26.3%	0.025*
Ventilator free days, *median (IQR) in days*	20 [14.5–23]	16.5 [0–21]	0.019*
PICU length of stay, *median (IQR) in days*	8 [5–13]	10 [7.75–19.25]	0.030*
MV duration, *median (IQR) in days*	8 [5–11.5]	9 [6.75–14.25]	*0.054*

A significant lower mortality was found in the PALICC population with unilateral consolidations (9.3%) compared to bilateral consolidations (26.3%). For the cohort with unilateral consolidations also a significant difference was found in VFD (median 20 vs 16.5 days) and PICU length of stay (median 8 vs 10 days) compared to bilateral consolidations. No significant difference was found for the MV duration

*Indicates significant differences

**Fig. 4:**
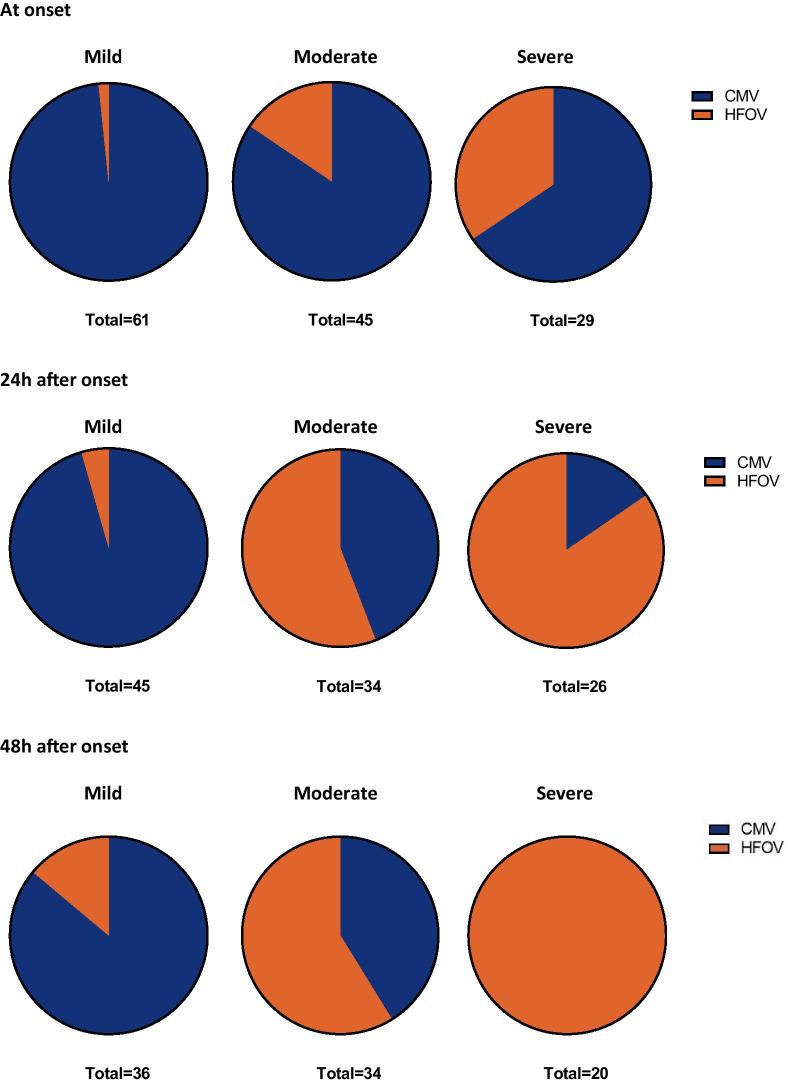
Distribution of ventilator mode stratified by PARDS severity at three time points (onset of PARDS, and 24 and 48 h after onset). The blue area represents CMV and the orange area HFOV. At onset 135 met PALICC criteria. 98% of the subjects with mild PARDS received CMV, 84.4% of the moderate group received CMV and 65.5% of the subjects meeting PALICC criteria for severe PARDS at onset received CMV. Twenty-four hours after onset still 105 met PALICC criteria (45 mild, 34 moderate, 26 severe). Of the group with mild PARDS 95.5% received CMV, 44.4% of the moderate group received CMV 24 h after onset and only 15.4% of the subjects with severe PARDS 24 h after onset received CMV. Forty-eight hours after onset 90 subject still met PALICC criteria for PARDS (36 mild, 34 moderate, 20 severe). Of the group with mild PARDS after 48 h 86.1% received CMV at that moment, of the moderate group 41.1% received CMV, of the severe group 0% received CMV

**Fig. 5:**
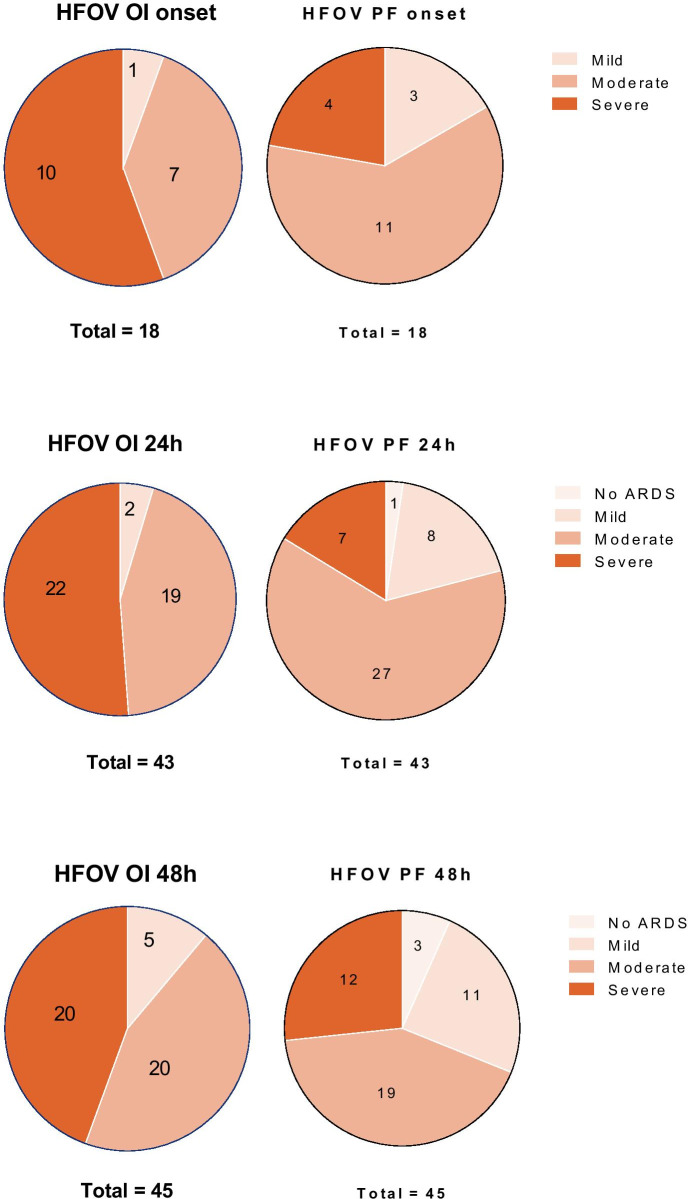
Difference in severity by oxygenation assessment method. Eighteen subjects received HFOV at PARDS onset. When severity was stratified by OI, 1 had mild PARDS, 7 moderate and 10 severe. When the PF is used for the same subjects 3 have mild ARDS, 11 moderate ARDS, 4 severe ARDS. Forty-three subjects received HFOV 24 h after onset, when severity is stratified by the OI 2 meet criteria for mild PARDS, 19 for moderate PARDS, 22 for severe PARDS. When the PF is used 1 doesn’t meet criteria for ARDS anymore, 8 meet criteria for mild ARDS, 27 for moderate and 7 for severe ARDS. Forty-five subjects received HFOV 48 h after onset. Hereof 5 met criteria for mild PARDS, 20 for moderate and 20 for severe PARDS. When these subjects were stratified by PF 3 do not meet ARDS criteria anymore, 11 meet criteria for mild ARDS, 19 for moderate ARDS and 12 meet criteria for severe ARDS

**Fig. 6:**
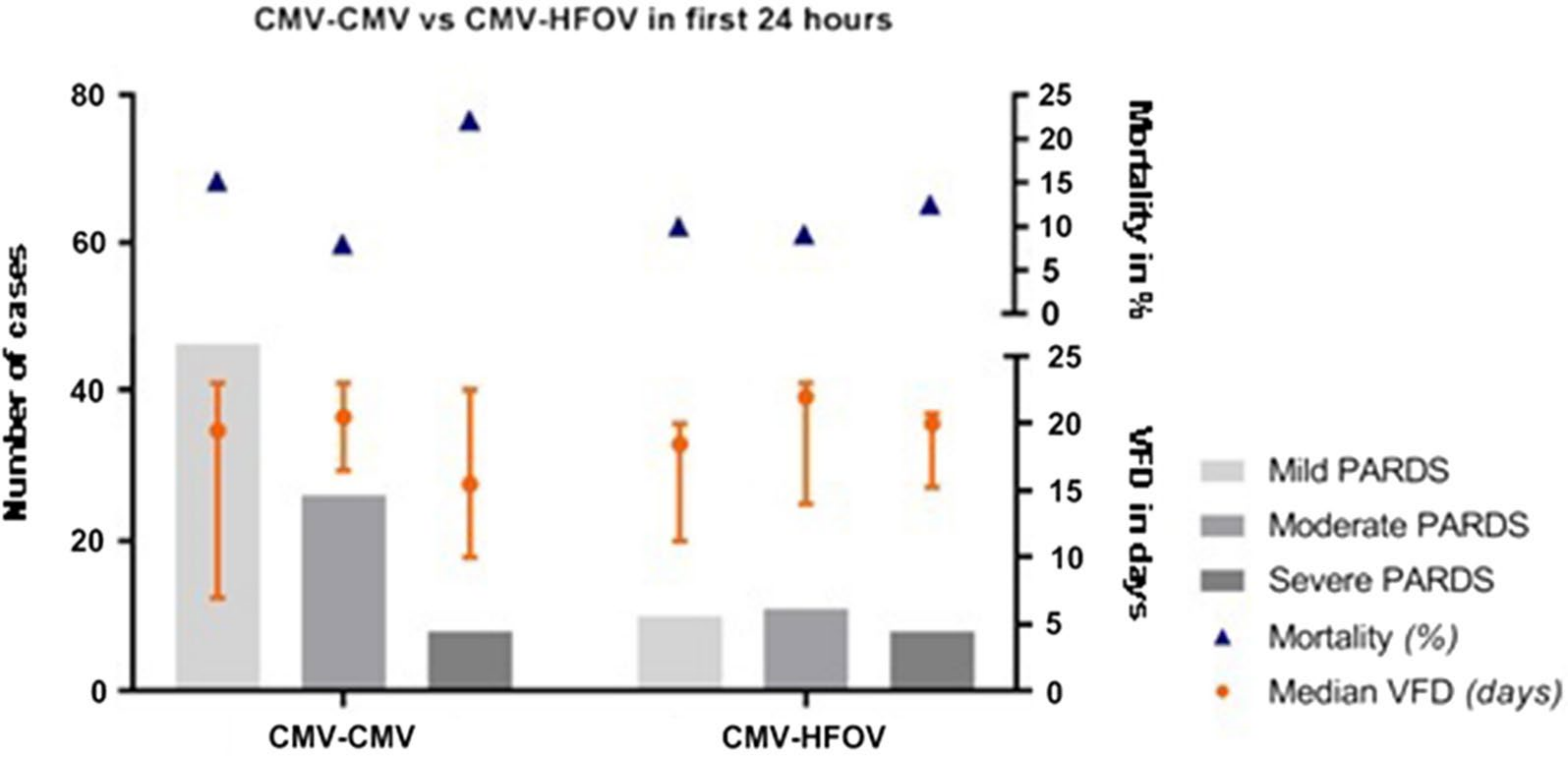
The effect of ventilation strategy in the first 24 h on the PARDS severity distribution, mortality and VFD. On the left the group of subjects receiving CMV the first 24 h after onset, on the right the group who are switched from CMV to HFOV within the first 24 h after onset. Grey bars represent number of cases with mild, moderate and severe PARDS. Orange dots depict median (with interquartile range) VFD-28 (bottom Y-axis) and blue triangles represent mortality (%) (upper Y-axis)

Eighteen patients received HFOV at PARDS onset, of whom 10 (55.6%) had severe PARDS (Fig. 4). At 48 h all patients with severe PARDS (N = 20) received HFOV. Application of the Berlin definition affected severity stratification (Fig. 5). Of the 117 patients receiving CMV at onset, 29 patients received HFOV 24 h after onset and 8 were extubated/deceased. VFD and mortality did not differ between the groups (Fig. 6). When patients were stratified in three categories by MV strategy over 48 h (HFOV for 48 h, partly HFOV/partly CMV and CMV for 48 h) there was no significant difference found in mortality (*p* = 0.833) nor in VFD (*p* = 0.135).

## Discussion

The main finding of this study is that identification of PARDS improved significantly when paediatric specific definition was applied. This was mainly due to not mandating bilateral consolidations on chest radiograph. Both existing adult definitions and the paediatric specific definition poorly stratified the risk for mortality in our cohort.

The PALICC PARDS definition was created to overcome the limitations of the adult-based definitions including epidemiological differences and the need for invasive metrics for oxygenation [18]. The international, observational Paediatric acute respiratory distress syndrome incidence and epidemiology (PARDIE) confirmed a better performance of PALICC PARDS in terms of identification of new PARDS cases and risk stratification [15]. The number of new PARDS cases in our study increased with the PALICC definition, comparable to PARDIE and numbers reported by others in retrospective single-center studies [19, 20]. However, in PARDIE less than half of the newly diagnosed PARDS cases was based on invasive metrics of oxygenation. Furthermore, there was a higher use of arterial blood gas in non-survivors, suggesting that there was a preference not to use indwelling arterial lines in lesser sick patients not being on vaso-active drugs as has been suggested previously [13]. In contrast, in our unit indwelling arterial lines are routinely inserted upon start of MV and not just for better haemodynamic monitoring, making that almost all our newly identified PARDS cases were based on arterial blood gasses. Also, our study differs from PARDIE as we did not include any patient on non-invasive ventilation (not offered in our unit). Like PARDIE, we observed the highest mortality rate among severe PARDS patients. Since our study was retrospective, it may thus be surmised that not the definition itself, but increased awareness may at least partially better identify new cases of PARDS.

We observed a prevalence of new PARDS of 5.5% which is in accordance with literature of the PALICC definition which ranges from 2.7 to 5.7% [15, 19, 20]. Our prevalence is most likely to be underestimated since patients who received NIMV, had home ventilation or cyanotic heart disease were excluded in this study although these are specially categorized in the PALICC definition.

PALICC PARDS poorly stratified risk of mortality in our study, contrasting the findings from the PARDIE study [15]. However, PARDIE only found higher mortality rates among patients with severe PARDS, an observation that was also made in the patient cohort coming from the Children’s Hospital of Los Angeles (CHLA) [19]. This lack of performance is not easily explained. From a methodological perspective, it might be argued that incorporating non-invasive metrics for oxygenation in the PALICC definition dilutes the cohort by including less sick PARDS patients, although in the PARDIE study the way how oxygenation was assessed (invasive vs non-invasive) did not appear to be associated with hypoxaemia severity. Alternatively, mortality may not be the right endpoint in PARDS since many patients die with and not from ARDS [21]. In fact, neurologic aetiologies of death in children with PARDS are common, confounding true mortality rates [22]. Overall mortality in our study was low, like other high-income countries but lower than low-income countries, and increased with increasing severity [23, 24]. We found that the best risk prediction for mortality, duration of MV, VFD and PICU length of stay was 24 h after onset by the PALICC definition, which is like the CHLA dataset but different from PARDIE reporting that this could be best done 6 h after PARDS diagnosis [15, 19]. Importantly, PARDS is a heterogenous syndrome with, calling for unpacking the syndrome by PARDS trigger or by clinical phenotype. For example, respiratory syncytial virus (RSV) lower respiratory tract infection may cause PARDS but is associated with low mortality [25]. Thus, future studies evaluating paediatric specific definitions for ARDS may consider stratifying by PARDS trigger, disease severity and cause of death.

Surprisingly, we found that not only PALICC but also the adult based Berlin definition performed poorly in terms of outcome stratification. De Luca and colleagues provided support for the usefulness of the Berlin definition in infants, but we could not confirm this in the present study [26]. However, we did find that the presence of bilateral consolidations on chest radiograph significantly added to mortality in contrast with the PARDIE study, making that there was higher mortality rate in severe patients when the Berlin definition was applied [27]. This warrants further study.

Our findings may also have been confounded by a liberal use of HFOV Patients receiving HFOV had more severe PARDS and all patients who met criteria for severe PARDS 48 h after onset received HFOV. In our unit, HFOV is used as an alternative mode of ventilation to prevent conventional ventilator settings becoming toxic or when there is severe hypoxaemia. Since we use an open-lung strategy characterised by high mPaw, automatically the OI will be higher and thereby influence the PARDS severity strata [28]. Interestingly, we observed lower mortality rates in patients managed with HFOV within 24 h after PARDS diagnosis, but numbers were too small to draw statistically significant conclusions.

There are various limitations to our analysis. First, our study was designed as a retrospective, single centre study. Although our unit probably is comparable to many other units based in high-income countries, our institutional practices including the times per date ABGs are done may vary confounding the main outcome parameters. Inherent with the retrospective design, clinical decision-making could not be captured, and missing data were not imputed. Second, pulse oximetry data were retrospectively making that precise alignment of mPaw, SpO_2_ and FiO_2_ could not be guaranteed; this has also been reported by others [29]. Furthermore, the number of patients with SpO_2_ > 97% precluded application of the PALICC criteria. Third, the presence of LV dysfunction was not evaluated in all patients and only deemed present if noted in the electronic health record. Lastly, evaluating chest radiographs is very subjective and has a proven high inter-observer variability [27, 30–32]. Our strategy was modelled after clinical practice where one observer interprets the chest-radiograph and only in case of uncertainty by a second observer. Inherently, this led to a high preselection and made it unreliable to calculate Cohen’s Kappa.

## Conclusions

This single-centre study confirmed that PALICC identified more new cases PARDS than the adult-based Berlin definition. However, both PALICC and Berlin performed poorly in terms of mortality risk stratification. The presence of bilateral consolidations was associated with a higher mortality rate. Our findings may be considered in future modifications of the PALICC criteria. They also indicate that patient with severe PARDS with bilateral consolidations may be the primary target population for future randomised controlled trials.

## Supplementary Information

**Additional file 1. **Flow chart of the experimental design. First all patients were selected who received MV over 24 h during PICU admission, then there was screened for exclusion criteria. Of the remaining cohort all chest radiographs were reviewed during day -1 up to day 8 of PICU admission by the first reviewer (MR). In case of a possible consolidation the second reviewer reviewed the chest radiographs (JvD). Hereof another selection was made and reviewed by a third reviewer (MK or RB), in case of a found consolidation the patient card was screened for the oxygenation criterium +/− 24 h of the chest radiograph.

## Data Availability

The datasets used and/or analysed during the current study are available from the corresponding author on reasonable request.

## Abbreviations

ABG: Arterial blood gas
AECC: American–European consensus conference
ALI: Acute lung injury
ARDS: Acute respiratory distress syndrome
AUC: Area under the curve
BD: Berlin definition
CDP: Continuous distending pressure
CI: Confidence interval
CMV: Conventional mechanical ventilation
CPAP: Continuous positive airway pressure
ECLS: Extracorporeal life support
HFOV: High frequency oscillatory ventilation
(I)MV: Invasive mechanical ventilation
IQR: Interquartile range
LV: Left ventricular
mPaw: Mean airway pressure
NIMV: Non-invasive mechanical ventilation
OI: Oxygenation index, formula: [FiO_2_ × Paw × 100]/PaO_2_
OSI: Oxygen saturation index, formula: [FiO_2_ × Paw × 100]/SpO_2_
PALICC: Paediatric acute lung injury consensus conference
PARDS: Paediatric acute respiratory distress syndrome
Paw: Mean airway pressure
PEEP: Positive end expiratory pressure
PF: PaO_2_/FiO_2_
PICU: Paediatric intensive care unit
PIM: Paediatric index of mortality
PRISM: Paediatric risk of mortality score
ROC: Receiver operating curve
RSV: Respiratory syncytial virus
UMCG: University Medical Center Groningen
VFD: Ventilator free days at 28 days
