# Supplementary figures and images for: Performance of acute respiratory distress syndrome definitions in a high acuity paediatric intensive care unit
Source: Respir Res. 2021 Sep 29;22:256. doi: 10.1186/s12931-021-01848-z (PMC8480111; doi:10.1186/s12931-021-01848-z)

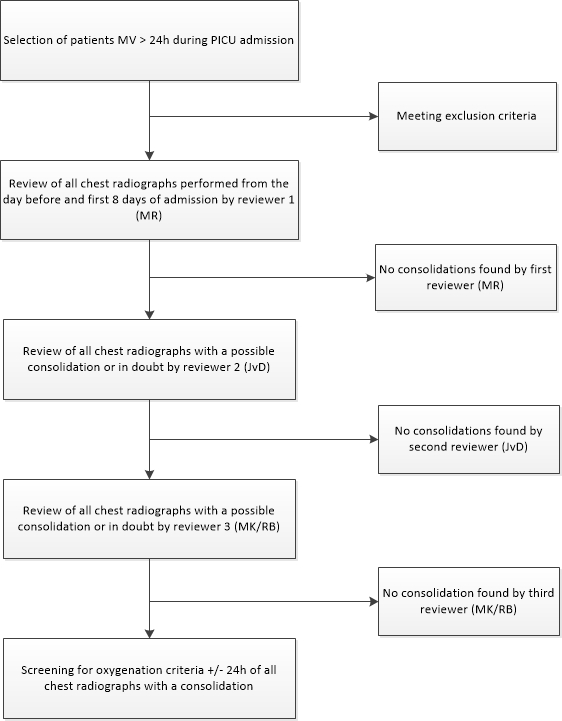

Supplement: Supplementary file 1 — Additional file 1. Flow chart of the experimental design. First all patients were selected who received MV over 24 h during PICU admission, then there was screened for exclusion criteria. Of the remaining cohort all chest radiographs were reviewed during day -1 up to day 8 of PICU admission by the first reviewer (MR). In case of a possible consolidation the second reviewer reviewed the chest radiographs (JvD). Hereof another selection was made and reviewed by a third reviewer (MK or RB), in case of a found consolidation the patient card was screened for the oxygenation criterium +/− 24 h of the chest radiograph. [file 12931_2021_1848_MOESM1_ESM.docx]
